# Перспективы создания Национального центра персонализированной медицины эндокринных заболеваний

**DOI:** 10.14341/probl12730

**Published:** 2021-01-26

**Authors:** И. И. Дедов, Н. Г. Мокрышева, М. В. Шестакова, П. Ю. Волчков, А. Ю. Майоров, О. Ю. Реброва, В. О. Барышева, Ю. А Крупинова, Е. А. Трошина, Л. К. Дзеранова, Г. А. Мельниченко

**Affiliations:** Национальный медицинский исследовательский центр эндокринологии; Национальный медицинский исследовательский центр эндокринологии; Национальный медицинский исследовательский центр эндокринологии; Национальный медицинский исследовательский центр эндокринологии; Национальный медицинский исследовательский центр эндокринологии; Национальный медицинский исследовательский центр эндокринологии; Национальный медицинский исследовательский центр эндокринологии; Национальный медицинский исследовательский центр эндокринологии; Национальный медицинский исследовательский центр эндокринологии; Национальный медицинский исследовательский центр эндокринологии; Национальный медицинский исследовательский центр эндокринологии

**Keywords:** персонализированная медицина, регенеративная медицина, клеточная терапия, генная терапия, эндокринология, генетика, биоинформатика, фармакогеномика, метаболомика, протеомика, клеточные технологии, радиотераностика

## Abstract

ФГБУ «НМИЦ эндокринологии» Минздрава России (НМИЦ эндокринологии) получил право реализовать программу развития Научного Центра Мирового Уровня «Национальный Центр Персонализированной медицины эндокринных заболеваний» (НЦПМЭЗ). Задачей НЦПМЭЗ станет не только создание системы персонализированного лечения, но и подготовка новых кадров для медицины. Фундаментальные исследования, проводимые на базе уже существующих институтов и лабораторий НМИЦ эндокринологии, будут расширены за счет создаваемых de novo лабораторий НЦПМЭЗ в соответствии с одобренным проектом.  Данная публикация знакомит читателя с наиболее значимыми лабораториями, которые планируется создать в НЦПМЭЗ: лабораторией общей, молекулярной и популяционной генетики, биоинформатики, фармакогеномики, микробиоты, редактирования генома, математических и цифровых технологий, неинвазивных технологий диагностики эндокринопатий, клеточных технологий, искусственного интеллекта и принципиально новой, не имеющей аналогов, лабораторией метаболической визуализации и радиотераностики.  Авторы надеются, что читатели одного из основных журналов для эндокринологов нашей страны примут активное участие в осуществлении планов НМИЦ эндокринологии, поскольку как у молодых, так и у опытных талантливых исследователей появится шанс стать частью команды Центра. Для выполнения амбициозных задач необходимо кардинально изменить мировоззрение врача в стране, разработать новую систему обучения за счет увеличения числа специалистов по медицинской генетике, транскриптомике, биостатистике и биоинформатике, работающих на стыке экспериментальной и клинической эндокринологии, что обеспечит транзит инновационных технологий в клиническую практику. Созданные лаборатории Научного Центра Мирового Уровня станут местом будничной работы нового поколения врачей, владеющих не только основами клинической работы, но и навыками фундаментальных исследований, что позволит им значительно улучшить методы прогнозирования, диагностики и лечения.

## Abstract (en)

The National Medical Research Center for Endocrinology (NMRCE) received the right to implement the development program of the World-class Research Centre “The National Center for personalized medicine of endocrine diseases” (NMCPMED). The objective of the NMCPMED will be not only the creation of a system of personalized treatment, but also the training of new specialists for medicine.  Fundamental researches, carried out on the basis of the already existing institutes and laboratories of the NMRCE will be expanded by creating new laboratories of the NCPMED created de novo in accordance with the approved project. This article introduces the reader to the most important laboratories that would be created in NCPMED. These are laboratories of general, molecular and population genetics, bioinformatics, pharmacogenomics, microbiota, genome editing, mathematical and digital technologies, non-invasive technologies for the diagnosis of endocrinopathies, cellular technologies, artificial intelligence and a fundamentally new laboratory of metabolic visualization and radioteranostics. The authors hope that readers of one of the main journals for endocrinologists in our country will actively participate in the  implementation of NMRCE, as both young and experienced talented researchers will have a chance to be a part of the Centre. To realize the ambitious implementation plans for the achievements of the Centre, it is necessary to radically change the worldview of the doctors in our country, to train them in a new way, and to expand the structure of the Center’s team by increasing the number of specialists in medical genetics, transcriptomics, biostatistics and bioinformatics, working at the intersection of experimental and clinical endocrinology, and ensuring the transit of innovative technologies into clinical practice. New laboratories of the World-Class Research Center, will become the place of routine work of a new generation of doctors, who possess not only the basics of clinical work, but also the skills of fundamental researches that will allow them to significantly improve the methods of diagnosis and treatment.

## ВВЕДЕНИЕ

В соответствии с указом Президента Российской Федерации от 7 мая 2018 г., №204  ФГБУ «НМИЦ эндокринологии» Минздрава России (далее — НМИЦ эндокринологии) как учреждение, совмещающее медицинскую, лечебную и научную деятельность с важнейшей педагогической функцией подготовки квалифицированных кадров для здравоохранения, получил право реализовать программу развития Научного Центра Мирового Уровня «Национальный Центр Персонализированной медицины эндокринных заболеваний» (далее — НЦПМЭЗ), представив на конкурс уникальное интегративное решение проблемы персонализации диагностики и лечения путем сопоставления геномных данных с метаболомными и гормональными параметрами в группах «природных моделей» первичных и вторичных нарушений в одной из основных интегративных систем организма — эндокринной системе. Задачей НЦПМЭЗ станет не только создание платформы для персонализированного лечения, но и подготовка новых кадров для медицины. 

Уникальный клинический 100-летний опыт НМИЦ эндокринологии обеспечил воспроизводимую систему обучения врачей технологиям диагностики различных эндокринопатий, от социально значимых (сахарный диабет (СД) в его различных формах с многообразными осложнениями и без них) до редчайших орфанных заболеваний. НМИЦ эндокринологии — многопрофильный исследовательский медицинский центр, не имеющий аналогов в мире, состоящий из семи институтов: диабета, клинической эндокринологии, детской эндокринологии, репродуктивной медицины, онкоэндокринологии, Института высшего и дополнительного профессионального образования, а также Института персонализированной медицины с межинститутскими лабораторными подразделениями. Основное внимание в научной и лечебной работе НМИЦ эндокринологии уделяется проблемам СД и его осложнениям, аутоиммунным и орфанным заболеваниям, опухолевым и гиперпластическим процессам эндокринных органов.

Особое значение имеет тот факт, что президент НМИЦ эндокринологии, академик РАН Иван Иванович Дедов, является основоположником персонализированного лечения эндокринопатий в России, за что был удостоен ­Государственной премии Российской Федерации в области науки и технологий за 2017 г. Фундаментальные исследования, проводимые на базе существующих институтов и лабораторий, будут расширены за счет создаваемых de novo лабораторий НЦПМЭЗ в соответствии с одобренным проектом. В настоящее время Институт Персонализированной медицины включает отделение патоморфологии со службой биобанкинга и криоконсервации, лабораторию генетики, лабораторию метаболомики и клеточных технологий.

Наиболее значимыми планируемыми новыми лабораториями являются лаборатории общей, молекулярной и популяционной генетики, биоинформатики, фармакогеномики, микробиоты, редактирования генома, математических, цифровых и инвазивных технологий диагностики эндокринопатий, клеточных технологий, искусственного интеллекта и принципиально новой, не имеющей аналогов лаборатории метаболической визуализации и радиотераностики. 

Очень важно, чтобы читатели одного из основных журналов для эндокринологов нашей страны как можно раньше узнали о планах создаваемого Центра и приняли активное участие в их осуществлении, поскольку как у молодых, так и у опытных талантливых исследователей появится шанс стать частью команды Научного Центра Мирового Уровня.

Для представления масштабов дальнейшего развития персонализированной терапии эндокринопатий мы приводим краткую характеристику каждой из планируемых в НЦПМЭЗ лабораторий, охватывающих самые перспективные направления.

1. В лаборатории общей молекулярной и популяционной генетики будут продолжаться новаторские исследования в области популяционной генетики СД 1 типа и диабета MODY, врожденной дисфункции коры надпочечников (ВДКН) и других эндокринопатий, в которых региональная гетерогенность распределения мутаций является основанием для селективных скринингов и позволяет воздействовать на образ жизни и планирование семьи с носителями известных герминальных мутаций, а также осуществлять поиск редких мутаций, накапливающихся в связи с «эффектом основателя». Не менее важными представляются исследования генетики синдромов множественных эндокринных неоплазий и нейроэндокринных новообразований, синдромов изолированных опухолей гипофиза, дайсеропатий (опухолей с мутацией в гене DICER), синдрома Карни и Мак Кьюна–Олбрайта, при котором преждевременное половое развитие у детей сочетается с опухолями эндокринной и других систем. Генетически детерминированные опухоли различной локализации создают серьезнейшую проблему ранней диагностики и лечения, которая может быть разрешена только с помощью новейших технологий геномной и метаболомной персонализации в сопоставлении с клинической картиной. Создание биобанка тканей, крови и библиотек (ДНК/РНК) позволит на основе уникального материала обеспечивать адресный скрининг ближайших родственников пробанда с подбором превентивного лечения, комбинирующего свойства таргетных препаратов со сродством к выявляемым в ткани опухоли рецепторам с радиоизотопной меткой. Устанавливая носительство онкогенных и иных наследуемых мутаций и исключая из циклов репродукции эмбрионы с наследственными патологиями, можно прервать цепочку передачи данных мутаций с использованием современных технологий и обеспечить рождение здоровых детей.

Более сложная задача установления роли геномных полиморфизмов в развитии таких распространенных эндокринопатий как синдрома поликистозных яичников, ожирения, СД 2 типа также может найти свое решение при использовании методов полногеномного анализа, что станет реальным прорывом в выборе таргетной терапии данных заболеваний.

2. В лаборатории клеточных технологий будут продолжаться передовые исследования в области регенеративной медицины и плюрипотентных стволовых клеток человека (эмбриональные стволовые клетки, индуцированные плюрипотентные стволовые клетки (iPS-клетки)). В отличие от общемирового уровня, в России работы по данному направлению ведутся лишь в нескольких лабораториях. При этом исследования по изучению фундаментальных механизмов развития эндокринопатий и разработке способов персонализированной генной и клеточной терапии как наиболее распространенных, так и орфанных эндокринных заболеваний, проводимые на базе лаборатории, уникальны для нашей страны. Уже сейчас рутинно используются современные методы получения и валидации пациент-специфичных iPS-клеток, методы генной инженерии для редактирования мутаций пациентов и получения аутологичных клеток с пониженной иммуногенностью, разрабатываются протоколы дифференцировки iPS-клеток в эндокринные гормонпродуцирующие клетки (бета-клетки поджелудочной железы, паратиреоидные клетки околощитовидных желез) для моделирования заболеваний пациентов in vitro и создания клеточных продуктов для персонализированной терапии СД, множественных эндокринных неоплазий, первичного гиперпаратиреоза, гипопаратиреоза и других эндокринопатий. В настоящее время на базе лаборатории создан и продолжает пополняться уникальный биобанк плюрипотентных персонализированных клеток пациентов с различными наследственными эндокринопатиями. В распоряжении лаборатории имеется новейшее оборудование для реализации исследований мирового уровня. Ввиду имеющихся у коллектива и Центра опыта и задела, а также принимая во внимание малочисленность отечественных разработок в области регенеративной медицины мирового уровня, основной задачей лаборатории в рамках реализации программы НЦПМЭЗ является трансляция новейших достижений фундаментальной мировой науки и разработок в области регенеративной медицины и клеточных технологий в практическую эндокринологию в России.

3. Лаборатория биоинформатики — новая для НМИЦ эндокринологии лаборатория, которая должна обеспечить индивидуальное прогнозирование течения болезни c помощью анализа геномных и метаболомных данных, в том числе в сопоставлении с данными по транскриптомным миРНК и эпигеномным маркерам, исследованным в тканях и в крови. Принципиально новые генетические исследования (NGS, SCS), эпигеномные и транскриптомные маркеры протеомики и метаболомики, биобанкирование операционного материала и биожидкостей в сочетании с богатым клиническим материалом и современными математическими подходами к их анализу позволят значительно продвинуться в разработке персонализированных подходов к ведению больных.

4. Лаборатория микробиоты необходима для изучения комплексной экосистемы симбиоза микроорганизмов человека, адаптированной к конкретным органам и системам тела. Около двух третей комменсальной микробиоты представлено в кишечнике, и это, по сути, максимальное представительство внешней среды в организме человека, активно взаимодействующее с организмом хозяина путем реализации многих метаболических функций, в первую очередь обмена липидов и глюкозы, продукции витаминов и энергии, регуляции насыщения. Помимо метаболического воздействия, кишечная микробиота провоцирует эпигенетические изменения ключевых генов, которые модулируют начало и прогрессирование заболеваний. К этим патологиям относятся онкологические, аутоиммунные и метаболические заболевания, такие как ожирение и СД 2 типа. В связи с растущей распространенностью метаболических нарушений в последние годы внимание исследователей сосредоточено на выяснении связи между эпигенетическими механизмами, микробным составом кишечника и их роли в индукции этих заболеваний. Понимание механизмов может изменить стратегию профилактики целого ряда заболеваний или их осложнений, а модификация состава микробиоты — стать основой лечения. Исследование микробиома при первичном экзогенном и синдромальном ожирении методом метагеномного ДНК-секвенирования позволяет получить новые сведения о механизмах нарушения микробиомного состава и путях его коррекции. Вариации в композитном составе микробиоты описаны при аутоиммунных заболеваниях кишечника, СД 1 типа и даже аутоиммунных тиреопатиях.

Гипотетически микробиота может играть антиканцерогенную и противовоспалительную роль, а дисбаланс соотношения различных частей микробиомной системы, возникший под влиянием пищи, антибактериального лечения (столь частого при осложняющихся инфицированием эндокринопатиях — гиперкортицизме, диабетической стопе), оперативных вмешательств, в том числе бариатрических, в свою очередь, изменяет эффективность действия инсулина. Изучение возможной индукции ремиссии метаболических заболеваний путем модифицирования кишечной микробиоты также станет предметом исследований данной лаборатории.

Планируемое создание биобанка микробиоты человека при различных обменных и аутоиммунных эндокринопатиях в сопоставлении с клиническими и генетическими параметрами заболеваний может стать уникальным источником информации о ранее малоизученном компоненте адаптации организма к болезни и ключом к новым способам лечения.

5. Новые перспективы откроет Лаборатория редактирования генома. Редактирование генома как вид генной инженерии, при котором может быть проведено направленное изменение (включение, удаление или замена) целевых фрагментов последовательности ДНК в геноме организма  с использованием специфических генетических конструкций и белков-эндонуклеаз, или «молекулярных ножниц», в частности, на основе системы CRISPR/Cas9, представляется чрезвычайно перспективным методом для разработки терапии ряда моногенных тяжелых эндокринных заболеваний, в первую очередь ВДКН и адренолейкодистрофии (с вариантом надпочечниковой недостаточности). В настоящее время концепция редактирования генома как инновационного направления, реализуемого с применением метода высокопроизводительного ДНК-секвенирования, нередко рассматривается как путь необходимого прямого генетического манипулирования практически во всех клетках организма in vivo.

В рамках работы лаборатории планируется совместное выполнение исследований, в первую очередь с лабораторией клеточных технологий, а также проведение экспериментальных разработок in vivo на модели дефицита 21 гидроксилазы (СYP21) по возможности редактирования генома при ВДКН.

6. В создаваемой Лаборатории иммунологии и аутоиммунных заболеваний исследование маркеров иммунного ответа и воспаления, дополненное интегративной оценкой с помощью математических моделей нейронных сетей взаимосвязи иммуновоспалительных, гормональных и метаболических нарушений в патогенезе эндокринных заболеваний на клеточном и молекулярном уровнях, позволит впервые реально объединить данные по двум основным интегративным системам, разработать и валидировать новые иммунологические, иммуногенетические и молекулярно-биологические методы персонализации ранней диагностики, прогнозирования течения и эффективности терапии заболеваний органов эндокринной системы. Основным направлением научно-практической деятельности лаборатории, в рамках реализации концепции трансляционной и персонифицированной медицины, будет являться разработка новых моно- и мультиплексных методов анализа иммунологических маркеров эндокринопатий.

Заболевания эндокринной системы, в развитии которых важную роль играют нарушения иммунного ответа (СД, тиреопатии, аутоиммунные полигландулярные синдромы, надпочечниковая недостаточность, офтальмопатия, нейроэндокринные опухоли, некоторые формы гипогонадизма и др.), относятся к числу наиболее тяжелых хронических болезней человека.

Объединение исследований в области иммунологии и эндокринологии — одна из самых заманчивых и перспективных идей, базирующаяся на твердом фундаменте уже имеющихся в НМИЦ эндокринологии наработок по получению рекомбинантных аутоантигенов щитовидной железы и выявлению иммуногенных эпитопов, созданию панели антител для диагностики латентных форм надпочечниковой недостаточности, посвященная еще одному гигантскому пласту эндокринологии на стыке с иммунологией: аутоиммунным эндокринным заболеваниям, спектр которых колеблется от распространенных (аутоиммунные поражения щитовидной железы имеют минимум 15% населения) до сравнительно редких, но крайне опасных полигландулярных синдромов (последовательно поражающих ряд эндокринных желез, а также неэндокринные органы). Новым направлением является прогнозирование и профилактика аутоиммунных поражений желез внутренней секреции при лечении так называемыми ингибиторами контрольных точек иммунного ответа различных злокачественных новообразований.

В настоящее время необходимо создание многокомпонентной системы прогнозирования иммунных (как моно-, так и полигенных) расстройств в ядерных семьях и популяции в целом, их предотвращения, раннего выявления и эффективной коррекции. В последнее десятилетие для определения биомаркеров эндокринных заболеваний применяют высокотехнологичные автоматизированные аналитические системы с использованием как «классических» моноплексных методов иммунного анализа (непрямая реакция иммунофлуоресценции, иммуноферментный анализ, иммуноблот, иммунонефелометрия, хемилюминесцентный иммунный анализ, радиоиммуноанализ), так и мультиплексных диагностических методов на основе ДНК-, РНК-, белковых и клеточных микрочипов, полимеразной цепной реакции, проточной цитометрии.

Разработанные в НМИЦ эндокринологии системы раннего выявления аутоиммунных поражений и диагностические панели для мультиплексного анализа на основе биочипа позволяют одновременно определять антитела к различным органам и тканям в одном биологическом образце. Эксклюзивные медицинские технологии биочипирования лиц с любой аутоиммунной патологией для поиска предрасположенности к другим аутоиммунным поражениям обеспечивают персонализированный клинико-иммунологический мониторинг и прогнозирование риска развития заболевания.

7. Лаборатория интеллектуальных математических технологий персонализации диагностики и прогнозирования.

Задачей лаборатории станет разработка персонализированных подходов к ведению пациентов с эндокринопатиями путем анализа больших объемов данных, имеющихся и планируемых к получению в НМИЦ эндокринологии, современными методами искусственного интеллекта. Ежегодно в центре лечение и/или диагностические процедуры получают более 150 тыс. больных из РФ и стран ближнего и дальнего зарубежья. Современные форматы электронного хранения клинических, лабораторных и инструментальных, в том числе визуализационных, данных лиц с верифицированными на современном уровне диагнозами и 10–20-летним мониторингом исходов заболеваний, объединенные с технологиями обработки больших данных («big data») и методами искусственного интеллекта, в том числе искусственными нейронными сетями высокой мощности (построенными на основе многослойных персептронов) и других современных методов машинного обучения, обеспечивают выявление комплексов прогностических маркеров течения заболевания, создание высокоточных прогностических моделей и подбор, как ключа к замку, программ персонализированного лечения и реабилитации.

8. Важное место займет Лаборатория неинвазивных технологий диагностики эндокринопатий, клэмп-технологий и фармакокинетики. Эндокринология как клиническая дисциплина базируется на исследовании показателей гормонального статуса в базальных условиях и в условиях функциональных проб, при этом имеется тесная связь между нагрузочными неинвазивными тестами, клэмп-технологиями и технологиями изучения всасывания, распределения, метаболизма и выведения лекарственных веществ. Клэмп-­технологии, в первую очередь гиперинсулинемический эугликемический клэмп, внедрены в практику НМИЦ эндокринологии с начала 1990-х гг. и стали основой современных технологий изучения биоэквивалентности препаратов инсулина. Широкое использование клэмп-технологий с другими молекулами (в первую очередь, кальцием) и лекарственными препаратами позволит расширить наши представления о роли рецепторов в фармакокинетике лекарственных веществ. Кроме того, планируется расширение спектра субстратов для оценки метаболитов лекарств и гормонов как за счет уже привычных биологических субстратов (­слюна, моча), так и за счет новых — выдыхаемый ­воздух, волосы.

9. Лаборатория фармакогеномики станет новым подразделением в НМИЦ эндокринологии. Одним из важных направлений исследований в рамках технологий персонализированной медицины является изучение влияния генома на индивидуальную переносимость и эффективность различных лекарственных препаратов. Основная задача фармакогеномики — изучение аллельных вариантов генов, определяющих особенности индивидуальных фармакокинетических и фармакодинамических характеристик организма с целью оптимизации стратегии лекарственной терапии.

Роль генетических факторов в индивидуальной реакции на терапию становится все более очевидной в последние годы, однако оценить терапевтический эффект лекарственного препарата в зависимости от индивидуальных генетических особенностей в ряде случаев довольно трудно.

Результаты полногеномного поиска ассоциаций (genome-wide association study, GWAS) позволили идентифицировать однонуклеотидные полиморфизмы (Single Nucleotide Polymorphism, SNP), ассоциированные с особенностями метаболизма и эффективности ряда препаратов. Фармакогенетические особенности известны для различных антидиабетических препаратов. При приеме метформина полиморфизм генов SLC22A1, PPARA, AMHR2, KCNJ11, SLC47A1, CAPN10 и др. может повышать/снижать эффективность терапии, полиморфизм гена SLC22A1 прогнозирует развитие желудочно-кишечных нежелательных лекарственных реакций (НЛР), SLC22A1, SLC22A2, SLC22A3 и др. меняют фармакокинетику. У производных сульфонилмочевины полиморфизм генов KCNJ11, CYP2C9, TCF7L2, ABCC8 и др. изменяет эффективность, SCNN1B, G6PD — повышает вероятность НЛР. При приеме глинидов полиморфизм SLCO1B1, IGF2BP2, KCNJ11, PAX4, KCNQ1 и др. влияет на эффективность, а SLCO1B1, NR1I2, CYP2C8, NEUROD1 и др. меняет фармакокинетические параметры. Эффективность ингибиторов дипептидилпептидазы-4 зависит от полиморфизмов генов GLP1R, CDKAL1, а лираглутида — от полиморфизма GLP1R. При лечении тиреотоксикоза носители 1 или 2 копий аллеля HLA-B имеют повышенный риск развития агранулоцитоза при приеме тиамазола, но не пропилтиоурацила, а при остеопорозе полиморфизм генов FDPS, VDR, MVK, FDPS меняет эффективность терапии бисфосфонатами, в то время как носительство полиморфизма гена CYP2C8 — предиктор риска остеонекроза челюсти на этом лечении. Как показали уже проведенные работы НМИЦ эндокринологии, эффективность лечения ожирения сибутрамином зависит от полиморфизма гена GNB3.

С помощью фармакогеномики может быть не только проведена персонализация лечения основного заболевания, но и предсказана эффективность либо вероятность развития осложнений при проведении терапии сопутствующих заболеваний, например, при приеме статинов, клопидогрела, ингибиторов протонной помпы и др.

Дальнейшее развитие и внедрение фармакогенетического тестирования в практику позволит разработать оптимальную индивидуальную стратегию лечения, подобрать наиболее эффективный препарат, режим дозирования, что снизит риск развития НЛР, повысит эффективность лечения и снизит стоимость проводимой терапии. Снижение риска развития НЛР и индивидуальный подход к терапии повысят комплаентность пациентов.

10. Лаборатория эмбриологии и сравнительной эндокринологии напрямую связана с работой отделения вспомогательных репродуктивных технологий, и на первых этапах планируется изучение особенностей сперматогенеза при ассоциированных с эндокринопатиями формах мужского бесплодия, обеспечение внутриматочной инсеминации и протоколов in vitro фертилизации (IVF) у лиц с эндокринопатиями, в том числе моногенными, криоконсервация спермы, работа с эмбрионами, оценка их морфологии, подбор оптимальных культуральных сред, научные исследования в области изоляции ооцитов и их идентификации, созревания и качества зигот; переноса эмбрионов, криоконсервации, совершенствование технологий микроманипуляций с гаметами или эмбрионами, включая интрацитоплазматические инъекции сперматозоидов, хетчинг и биопсию эмбриона.

11. Лаборатория молекулярной онкоэндокринологии станет важнейшим отделом НЦПМЭЗ, позволяющим выявлять генетические предикторы агрессивного поведения различных опухолей, от распространенных опухолей щитовидной железы, в том числе фолликулярных неоплазий с неопределенным злокачественным потенциалом (мутации BRAF, TERT, RET/PTC, RAS, PIK3CA, PAX8/PPARG, EGFR, CTNNB1, TP53), до симпато-адреналовых опухолей — феохромоцитом/параганглиом (SDH, VHL, RET, NF1, MAX, TMEM12) или синдромов множественных эндокринных опухолей 1 типа (MEN1) и 2 типа (RET). Ярким примером реализации принципов персонализированной медицины на основе генетических предикторов является выбор времени профилактической тиреоидэктомии (полного удаления щитовидной железы) у детей из семей с синдромом множественной эндокринной неоплазии 2 типа в зависимости от выявленной мутации (максимальный риск при мутации в кодоне М918, тиреоидэктомия выполняется в первые годы или даже первые месяцы жизни, высокий — при мутации в кодонах С634 и A883, умеренный — при мутациях в других кодонах, когда возможно наблюдение за гормональными маркерами, начиная с 5 лет, а проведение тиреоидэктомии, в случае выявления повышенного уровня кальцитонина, проводится в юности или у взрослых). Агрессивные аденомы гипофиза, рак околощитовидных желез требуют принципиально новых подходов лечения и диагностики, предикторы развития которых должны выявляться самыми передовыми методами.

12. Центр имеет собственные наработки в области радионуклидной диагностики и терапии, а также эксклюзивные разрешения на работу с радиофармпрепаратами для молекулярной визуализации и терапии — тераностиками. В 2019 г. получены санитарное эпидемиологическое заключение Роспотребнадзора и лицензия Ростехнадзора на эксплуатацию источников 177Lu, 223Ra и 225Ac, обладающих терапевтической способностью.

Создание Лаборатории метаболической визуализации и радиотераностики обусловлено необходимостью развития в нашей стране радионуклидных методов диагностики и лечения эндокринопатий. Совмещение терапевтической и диагностической функций (тераностика = терапия + диагностика) расширяет спектр имеющихся и позволяет разрабатывать новые радиофармацевтические лекарственные препараты (на основе метаболитов, пептидов, антигенов и антител) для диагностики и лечения патологических процессов. Радиотераностика объединяет радионуклидную диагностику (молекулярную визуализацию), дозиметрическое моделирование и радионуклидную терапию. Если вырабатывающая гормоны ткань имеет известную молекулярную мишень (как правило, мембранный белок-рецептор), то использование меток превращает одну и ту же молекулу, специфически накапливающуюся внутри или на поверхности патологических клеток-мишеней, и в средство ранней диагностики, и в таргетный терапевтический препарат.

## ЗАКЛЮЧЕНИЕ

Для реализации амбициозных планов Научного Центра Мирового Уровня и активного внедрения его возможностей и разработок в научный и лечебный процессы необходимо кардинально изменить мировоззрение врача в стране, разработать новую систему обучения за счет увеличения числа специалистов по медицинской генетике, транскриптомике, биостатистике и биоинформатике, работающих на стыке экспериментальной и клинической эндокринологии, что обеспечит транзит инновационных технологий и алгоритмов в клиническую практику. В настоящее время на базе Института высшего и дополнительного профессионального образования НМИЦ эндокринологии создана новая кафедра Персонализированной и трансляционной медицины.

Запланированные лаборатории Научного Центра Мирового Уровня станут местом будничной работы нового поколения врачей, владеющих не только основами клинической работы, но и навыками фундаментальных исследований, что позволит им значительно улучшить методы диагностики и лечения, а также обеспечит переход от унифицированной к персонализированной медицине. Все полученные научные и практические достижения НМИЦ эндокринологии как в прошлом, настоящем, так и в будущем вкупе будут направлены на непрерывное повышение качества, безопасности и эффективности новейших методов лечения для помощи каждому больному. 

## ДОПОЛНИТЕЛЬНАЯ ИНФОРМАЦИЯ

Участие авторов. Все авторы одобрили финальную версию статьи перед публикацией, выразили согласие нести ответственность за все аспекты работы, подразумевающую надлежащее изучение и решение вопросов, связанных с точностью или добросовестностью любой части работы.

Конфликт интересов. Авторы декларируют отсутствие явных и потенциальных конфликтов интересов, связанных с публикацией настоящей статьи.

Источники финансирования. Исследование выполнено при поддержке гранта Министерства науки и высшего образования Российской Федерации (соглашение № 075-15-2020-899). 

